# Identification of a lineage specific zinc responsive genomic island in *Mycobacterium avium* ssp. *paratuberculosis*

**DOI:** 10.1186/1471-2164-15-1076

**Published:** 2014-12-06

**Authors:** Elke Eckelt, Michael Jarek, Cornelia Frömke, Jochen Meens, Ralph Goethe

**Affiliations:** Institute for Microbiology, Department of Infectious Diseases, University of Veterinary Medicine Hannover, Bischofsholer Damm 15, 30173 Hannover, Germany; Genome Analytics, Helmholtz Centre for Infection Research, Braunschweig, Germany; Department of Biometry, Epidemiology and Information Processing, WHO Collaborating Centre for Research and Training in Veterinary Public Health, University of Veterinary Medicine, Hannover, Germany

**Keywords:** Zinc homeostasis, FurB, Zur, Regulation, Mycobacteria, Metalloregulator

## Abstract

**Background:**

Maintenance of metal homeostasis is crucial in bacterial pathogenicity as metal starvation is the most important mechanism in the nutritional immunity strategy of host cells. Thus, pathogenic bacteria have evolved sensitive metal scavenging systems to overcome this particular host defence mechanism. The ruminant pathogen *Mycobacterium avium* ssp. *paratuberculosis* (MAP) displays a unique gut tropism and causes a chronic progressive intestinal inflammation. MAP possesses eight conserved lineage specific large sequence polymorphisms (LSP), which distinguish MAP from its ancestral *M. avium* ssp. *hominissuis* or other *M. avium* subspecies. LSP14 and LSP15 harbour many genes proposed to be involved in metal homeostasis and have been suggested to substitute for a MAP specific, impaired mycobactin synthesis.

**Results:**

In the present study, we found that a LSP14 located putative IrtAB-like iron transporter encoded by *mpt*ABC was induced by zinc but not by iron starvation. Heterologous reporter gene assays with the *lac*Z gene under control of the *mpt*ABC promoter in *M. smegmatis* (MSMEG) and in a MSMEG∆*fur*B deletion mutant revealed a zinc dependent, metalloregulator FurB mediated expression of *mpt*ABC via a conserved mycobacterial FurB recognition site. Deep sequencing of RNA from MAP cultures treated with the zinc chelator TPEN revealed that 70 genes responded to zinc limitation. Remarkably, 45 of these genes were located on a large genomic island of approximately 90 kb which harboured LSP14 and LSP15. Thirty-five of these genes were predicted to be controlled by FurB, due to the presence of putative binding sites. This clustering of zinc responsive genes was exclusively found in MAP and not in other mycobacteria.

**Conclusions:**

Our data revealed a particular genomic signature for MAP given by a unique zinc specific locus, thereby suggesting an exceptional relevance of zinc for the metabolism of MAP. MAP seems to be well adapted to maintain zinc homeostasis which might contribute to the peculiarity of MAP pathogenicity.

**Electronic supplementary material:**

The online version of this article (doi:10.1186/1471-2164-15-1076) contains supplementary material, which is available to authorized users.

## Background

Transition metals such as iron and zinc are key factors in numerous biological processes as both, structural and catalytic cofactors for proteins. Therefore, they are important for the metabolic homeostasis and viability of eukaryotic and prokaryotic cells [1, 2]. However, high intracellular amounts of free iron and zinc also bear a great toxic potential. Consequently, cells have established sensitively regulated processes for maintaining a balanced intracellular metal homeostasis.

In mycobacteria, several importer, exporter and scavenger proteins as well as different regulator proteins have been described to contribute to metal homeostasis [3, 4]. The most important regulator of iron homeostasis in mycobacteria is the iron dependent repressor protein IdeR, a metal binding transcriptional regulator of the Diptheria-toxin Repressor (DtxR) family [5]. IdeR is a regulator with complex functions, on the one hand acting as a repressor of mycobactin synthesis and iron siderophore uptake transporters [6] and on the other hand it activates the synthesis of iron storage proteins. In addition, IdeR is involved in virulence and in the regulation of stress response and central metabolism [7]. The role of another regulator, the staphylococcal iron regulator repressor SirR in mycobacterial iron metabolism has been suggested, but has not been confirmed yet [8].

The maintenance of zinc homeostasis in mycobacteria is regulated by the metalloregulator system SmtB-FurB. Both regulators are co-transcribed from one operon, but work antagonistically. SmtB is a metal sensor of the ArsR-family, which senses free zinc ions and regulates the majority of zinc export systems [9], whereas the FUR family protein FurB, also known as Zur, is a zinc dependent repressor of zinc uptake systems [10–12].

*Mycobacterium avium* ssp. *paratuberculosis* (MAP) is the causative agent of paratuberculosis (Johne’s disease), a chronic, incurable enteritis in ruminants. MAP belongs to the *M. avium* complex (MAC). The MAC comprises genetically closely related subspecies with different extents of environmental adaptation and virulence. For instance, the MAC ancestral *M. avium* ssp. *hominissuis* (MAH) retained genes for environmental lifestyle, but can also cause opportunistic infections in humans, pigs and ruminants [13] *.* In contrast, MAP, as an obligatory pathogen, is unable to multiply in the environment [14] and needs the host for multiplication. Hence, MAP developed a particular tropism to the intestine [15], characterized by strong metabolic adaptations [16, 17], allowing massive MAP proliferation in the host at late stages of disease. The phenotypical differences in the MAC are associated with irreversible insertions and deletions of genomic fragments, so called large sequence polymorphisms (LSP) [18]. Thus, the MAP genome comprises seven exclusive lineage specific genomic insertions (LSP2, LSP4, LSP11, LSP12, LSP14, LSP15, LSP16) and one deletion (LSP8). Interestingly, several genes located on these LSPs have recently been found to be associated with virulence [19, 20].

A common feature of the pathogenicity of MAP and other pathogenic mycobacteria is the ability to persist intracellularly in macrophages by inhibiting the phagosomal maturation [21], thereby evading or contemporarily modulating host cell defence mechanisms [22–24]. Additionally, mycobacteria are able to overcome the so called nutritional immunity, a process induced by host cells and characterized by microbial nutrient starvation or intoxication [1, 25–27]. Thus, macrophages are able to deplete essential iron ions by secreting scavenging chelating compounds such as haem, lactoferrin and ferritin. Furthermore, they are able to create an iron depleted environment in the phagosome by the activity of the natural resistance associated membrane protein (NRAMP) transporter [28, 29]. Most pathogenic mycobacteria are able to counteract iron starvation by the inducible expression of the iron chelating siderophores mycobactin and carboxymycobactin, the expression of which is controlled by IdeR [30].

MAP is unable to produce functional mycobactins. Even though the gene cluster for mycobactin synthesis is present in the genome, the genes *mbt*A and *mbt*E are truncated [31] which disturbs mycobactin production. Hence, MAP requires substituting systems. The **m**ycobacterium **p**aratuberculosis **t**ransporter gene cluster (*mpt*) is a promising candidate as it is predicted to encode two putative transporters (*mpt*ABC and *mpt*DEF) involved in metal transport [32, 33]. It is located on the MAP specific LSP14 along with two clusters encoding a putative siderophore synthesis system (*sid*, *map*3739c-3745) and a putative siderophore uptake system (*fep*, *map*3726-3728). In addition, the *mpt*ABC operon shows homologies to the iron uptake transporter IrtAB of *M. tuberculosis* (Mtb) [33]. In Mtb, IrtAB was shown to contribute to virulence and maintenance of iron homeostasis by mediating mycobactin uptake [6, 33].

Despite the need of MAP for an iron substituting system, the function and the regulation of the genes of LSP14 and LSP15 is unclear. Regulation by IdeR is unlikely as no binding site for this regulator could be found in the promoter region [34]. Furthermore, the ferric uptake regulator FurA has recently been shown to be not involved in the regulation of the genes of LSP14 and LSP15 (Eckelt et al., unpublished observations).

Since the maintenance of metal homeostasis is crucial for survival of MAP in the host, we were interested to elucidate the role of LSP14 and LSP15 in metal homeostasis. In the presented work, we found that the *mpt*ABC transporter is regulated by zinc and that the transcriptional regulator FurB is directly involved in the zinc dependent regulation of *mpt*ABC. Furthermore, we analysed the global response of MAP to zinc starvation and were able to identify a unique zinc responsive genomic island in MAP.

## Results

### Metal dependent regulation of a MAP specific genome region

The MAP specific LSP14 and LSP15 harbour many genes proposed to be involved in metal homeostasis [18, 32]. Amongst these *map*3736c-3734c (*mpt*ABC) encode for an IrtAB like transporter [6, 33], the gene cluster *map*3739c-3745 (*sid*) for a siderophore synthesis system and *map*3726-3728 (*fep*) for another predicted siderophore uptake system. Therefore, we were interested whether these genes were regulated metal dependently. To investigate this, we exposed MAP to different metal starvation conditions and analysed changes in gene expression by qRT-PCR. Iron starvation was achieved by addition of the cell membrane permeable iron chelator 2,2-bipyridyl (DIP), thereby depleting intracellular Fe^2+^ ions. In these experiments, the *mbt*B gene (*map*2177c) of the mycobactin cluster, which is known to be induced by iron starvation in Mtb and MAP [35, 36], was included as positive control.

**Figure 1:**
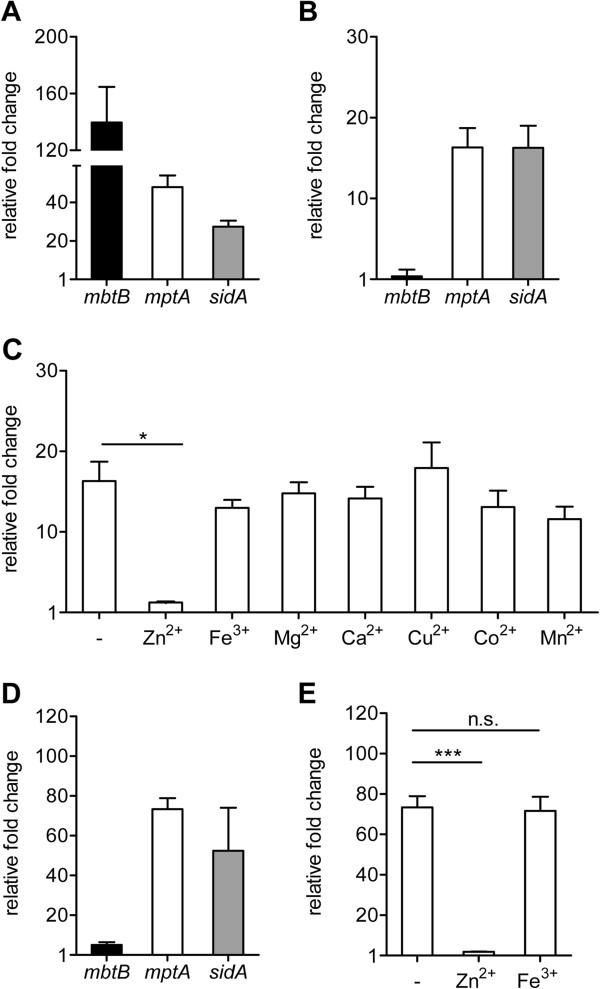
**Metal dependent regulation of a** ***M. avium*** **ssp.** ***paratuberculosis*** **specific gene locus.** MAPwt was grown in MB-complete to an OD_600_ of 1.0 and treated with different chelating agents and supplements as described in Methods. After RNA extraction, changes in gene expression levels of *mbt*B (black bars), *mpt*A (white bars) and *sid*A (grey bars) were analysed by qRT-PCR. **(A)** 200 μM 2,2-bipyridyl (DIP) for 2 h. **(B)** 14 mM nitrilotriacetic acid (NTA) for 24 h. **(C)** NTA treated cultures (14 mM, 24 h) supplemented with 1 mM ZnSO_4_, FeSO_4_, MgCl_2_, CaCl_2_, CuSO_4_, CoCl_2_ or MnSO_4_. **(D)** 10 μM N,N,N′,N′-tetrakis (2-pyridylmethyl) ethylenediamine (TPEN) for 2 h. **(E)** TPEN treated cultures (10 μM, 2 h) supplemented with ZnSO_4_ or FeSO_4_ both in a final concentration of 7.5 μM. Shown are the results of at least three independent experiments (mean ± SEM). Results were normalized to the housekeeping gene *gap* and are expressed as fold change compared to the untreated controls. Statistical analyses were performed using Kruskal-Wallis test (C) with *p < 0.01 and ***p < 0.0001 or Mann–Whitney test (E) with ***p < 0.0001.

As shown in Figure 1A, compared to the untreated control, expression of *mbt*B was significantly induced upon DIP treatment (200 μM final), indicating successful iron chelation. Expression of *mpt*A and *sid*A, representing the *mpt*ABC and *sid*-operon, respectively, was also enhanced after DIP treatment, however, to a considerably lower extent than *mbt*B. To dissect whether this induction was due to iron starvation or DIP chelation of other metal ions, we analysed the expression of these genes in cultures treated with nitrilotriacetic acid (NTA) at a final concentration of 14 mM. NTA is a less selective non permeable chelator which binds Fe^3+^ and many other metal ions. As shown in Figure 1B, treatment of MAP cultures with NTA, even over extended time, did not affect *mbt*B expression, whereas *mpt*A and *sid*A were still induced. The *fep* cluster responded neither to DIP nor to NTA treatment (data not shown). These results suggested that metal ions other than iron are involved in the regulation of the *mpt*ABC and *sid*-operon.

To evaluate this, we supplemented NTA cultures (14 mM final) with ZnSO_4,_ FeSO_4_, MgCl_2_, CaCl_2_, CuSO_4_, CoCl_2_ or MnSO_4_ each to a final concentration of 1 mM [37]. Compared to cultures treated with NTA only, induction of *mpt*A (Figure 1C) and *sid*A (data not shown) were significantly repressed only upon supplementation with ZnSO_4_, indicating a zinc dependent regulation of the *mpt*ABC and *sid*-operon. The specificity of a zinc dependent regulation was further investigated by exposure of MAP cultures to 10 μM N,N,N′,N′-tetrakis (2-pyridylmethyl) ethylenediamine (TPEN), a cell permeable chelating agent, which specifically binds intracellular zinc with high affinity. As expected, TPEN treatment resulted in a significant induction of *mpt*A and *sid*A, whereas expression of *mbt*B was only very slightly affected (Figure 1D). Vice versa, supplementation of TPEN treated cultures (10 μM final) with ZnSO_4_ but not FeSO_4_ (both in a final concentration of 7.5 μM) completely abolished *mpt*A induction (Figure 1E). In fact, titration experiments for the determination of the optimal concentration showed that the addition of 1 μM ZnSO_4_ (final concentration) was already sufficient to reduce induction of *mpt*A to approx. 50%, which emphasizes the high sensitivity of the regulation of *mpt*A by zinc (Additional file 1: Figure S5). Together these results demonstrated the zinc dependent regulation of the *mpt*ABC- and *sid*-operon.

### A functional FurB binding site is necessary for *mpt*ABC operon expression

**Figure 2:**
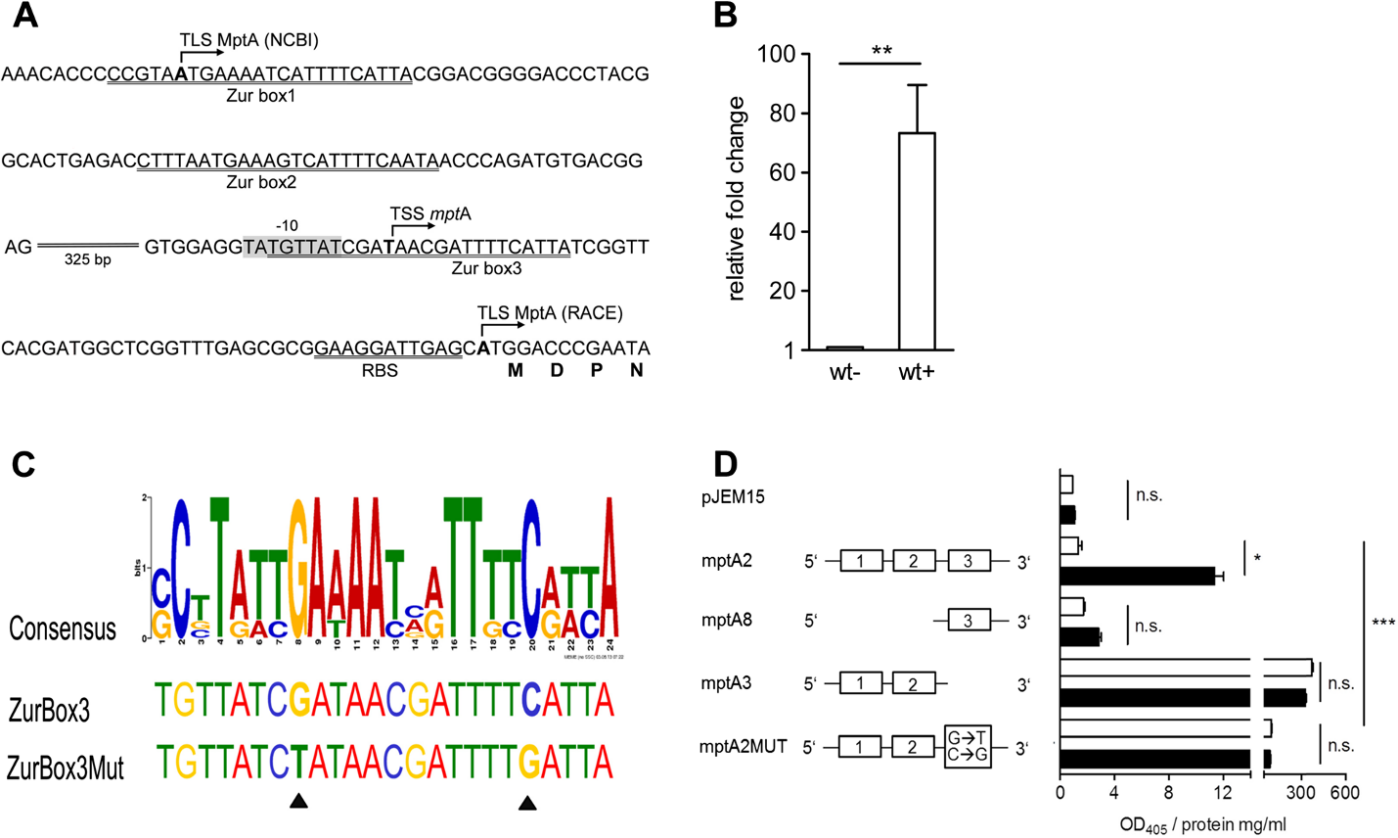
**Organisation and Zur dependent regulation of a MAP specific ABC transporter. (A)** Analysis of the *mpt*ABC promoter. MAPwt was grown in MB-complete to an OD_600_ of 1.0 and treated 2 h with 10 μM TPEN. Transcription start sites (TSS) were determined by 5’RACE. Depicted is the putative organisation of the *mpt*ABC promoter region [NCBI:NC_002944] (position 4158368 to 4158826). TSS and putative translation start sites (TLS) according to NCBI (NCBI) and 5’RACE results (RACE) are indicated in bold. A putative −10 promoter site is highlighted grey, putative Zur boxes and a ribosome binding site (RBS) are underlined. **(B)** Heterologous expression and regulation of the *mpt*ABC operon in MSMEG*.* MSMEG was transformed with pMP1102, cultured in MB-complete and treated with TPEN as described above. Gene expression of *mpt*A was analysed by qRT-PCR. Bars represent the relative fold change of the treated transformant (wt+) to the untreated control (wt-) (three independent experiments, mean ± SEM). Statistical analysis was performed using Mann–Whitney test with **p < 0.005. **(C)** Analysis of Zur binding sites in MAP by FIMO analysis. Upper panel: consensus sequence of Mtb-Zur [10] used for FIMO. Middle panel: Zur box3 of *mpt*A. Lower panel: mutated *mpt*A Zur box3, black arrows indicate mutated nucleotides. **(D)** Zur box analysis of the *mpt*ABC operon by β-galactosidase assay. MSMEGwt was transformed with the indicated *lac*Z-reporter plasmids: *mpt*A2, *mpt*A8, *mpt*A3 and *mpt*A2-MUT. Strains were grown in MB-complete and treated with TPEN as described above (black bars) or left untreated (white bars). Proteins were extracted and promoter activity was analysed by β-galactosidase assay (three independent experiments, mean ± SEM). Activity was measured at a wavelength of 405 nm and related to mg protein per ml. Statistical analysis was performed by using the Kruskal-Wallis-Test with *p >0.01 and ***p >0.0001.

Next, we were interested to elucidate the mechanisms of zinc dependent regulation of the *mpt*ABC operon. First, we determined the transcriptional start site (TSS) of *mpt*A by 5’RACE from TPEN treated cultures and defined the promoter-operator elements as shown in Figure 2A. The TSS was located at nucleotide 4158897 [NCBI:NC_002944] and a putative translation start (TLS) codon [ATG] of *mpt*A was found +56 bp downstream (position 4158839 to 4158841), preceded by a putative ribosome binding site (RBS) GAAGGATTGAG (position 4158843 to 4158853). A putative −10 promoter element (TAT**G**TTAT) could be identified −8 bp from the TSS (position 4158901 to 4158908). Furthermore, we could identify three sequence motifs in the 5’ proximal promoter-operator region with high homology to the binding site of the zinc dependent metalloregulator FurB (Zur) of Mtb [10], hereafter designated as Zur box 1–3.

Following, we tested the ability of *Mycobacterium smegmatis* (MSMEG) to serve as a heterologous expression system by transforming MSMEG with the plasmid pMP1102 [38], harbouring the complete *mpt* cluster and 941 bp upstream the TSS of *mpt*A. Then, cultures were left untreated or treated with TPEN at a final concentration of 10 μM and expression of *mpt*A was analysed by qRT-PCR. As shown in Figure 2B, we observed a clear induction of expression upon zinc starvation compared to the untreated control. This indicated that the regulation of *mpt*ABC in MSMEG was similar to that in MAP, thereby demonstrating the suitability of the system. To elucidate the relevance of the Zur boxes we cloned 984 bp from −941 to +42 relative to the TSS of *mpt*A, harbouring all three putative Zur boxes but lacking the RBS, into the *lac*Z-reporter plasmid pJEM15. Next, we introduced the resulting plasmid pJEM-*mpt*A2 and the promoter-less pJEM15 vector into MSMEG and analysed β-galactosidase activity of untreated or TPEN treated cultures. As shown in Figure 2D, without TPEN treatment, both MSMEG transformants showed only low β-galactosidase activity similar to the insert-free pJEM15 transformant. Treatment with TPEN significantly increased β-galactosidase activity of MSMEG transformed with pJEM-*mpt*A2. Deletion of the two proximal Zur boxes (pJEM-*mpt*A8) abolished TPEN induced reporter activity, suggesting that additional upstream promoter elements are necessary for full *mpt*A regulation. On the other hand, deletion of the putative promoter elements including Zur box3 (pJEM-*mpt*A3) resulted in a complete loss of repression. To dissect the relevance of Zur box3, we generated pJEM-*mpt*A2MUT with two point mutations in Zur box3 (Figure 2C). By site directed mutagenesis we exchanged nucleotides G→T and C→G at positions 4158899 and 4158887 in pJEM-*mpt*A2 [TGTTATC**G**→**T**ATAACGATTTT**C**→**G**ATTA]. These mutations resulted in a complete loss of *mpt*A repression (Figure 2D), clearly showing a direct involvement of Zur box3 in regulation of the *mpt*ABC operon.

### The *mpt*ABC operon of MAP is regulated by FurB

**Figure 3:**
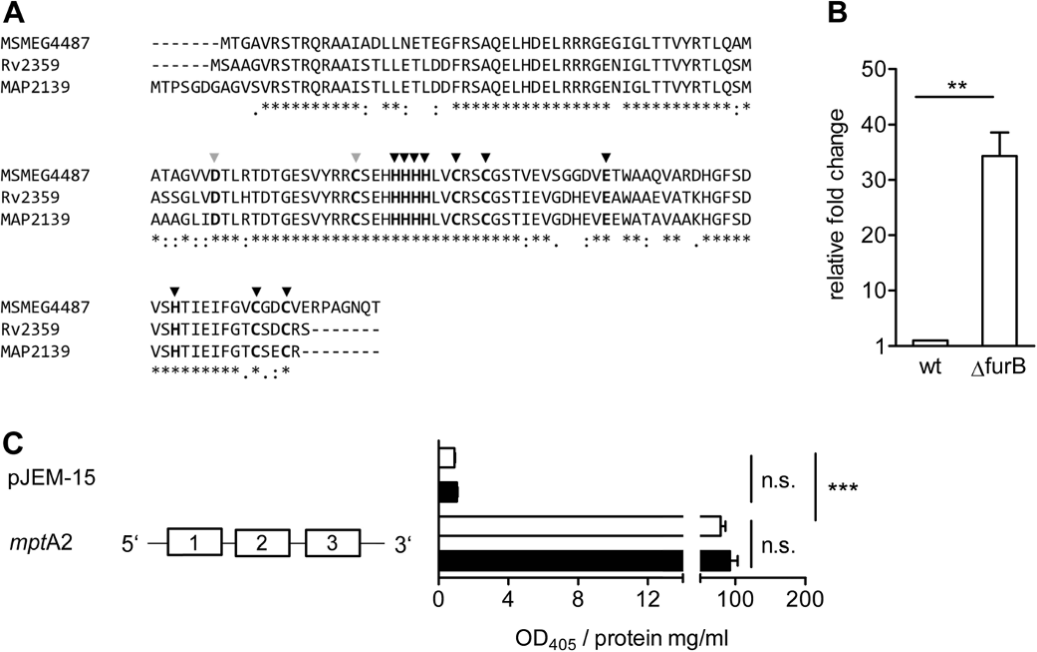
**Analysis of** ***mpt*** **A regulation by FurB by heterologous expression in** ***M. smegmatis*** **∆** ***fur*** **B (MSMEG∆** ***fur*** **B). (A)** FurB amino acid sequences of MAP, Mtb and *M. smegmatis* (MSMEG) were compared using ClustalOmega multiple sequence alignment. Asterices indicate homologue amino acids, grey arrows show highly conserved functional sites, black arrows structural sites (according to [39]). **(B)** MSMEG∆*fur*B was transformed with pMP1102, grown in MB-complete to an OD_600_ of 1.0 and gene expression of *mpt*A compared to MSMEG wildtype (wt) was analysed by qRT-PCR. Shown are the results of three independent experiments expressed as the relative fold change of gene expression of the ∆*fur*B mutant to the wildtype, normalized to the housekeeping gene *gap*. Statistical analysis was performed using Mann–Whitney test with **p < 0.005. **(C)** MSMEG∆*fur*B was transformed with pJEM15 or pJEM-*mpt*A2, grown in MB-complete to an OD_600_ of 1.0 treated with 10 μM TPEN for 2 h, proteins were extracted, concentration was determined and promoter activity of TPEN treated (black bars) and untreated cultures (white bars) was analysed by β-galactosidase assay. Results of at least three independent experiments (mean ± SEM) are shown. Activity was measured at a wavelength of 405 nm and related to mg protein per ml. Statistical analysis was performed by using the Kruskal-Wallis-Test with ***p >0.0001.

The above analysis suggested that Zur box3 mediated FurB repression of *mpt*A. Comparison of amino acid sequences of FurB of MAP [NCBI:NP_961073 = MAP2139] and MSMEG [NCBI:YP_888759 = MSMEG4487] to Mtb [NCBI:NP_216875 = Rv2359] by blastp and Clustal Omega analyses revealed high homologies of 90% and 80%, respectively, indicating a strong conservation within mycobacterial FurB proteins. Moreover, all catalytic and structural important amino acids [39] were highly conserved among the species (Figure 3A).

To prove FurB as the responsible regulator of *mpt*A, we deleted *fur*B (*msmeg*4487) in MSMEG and transformed the resulting mutant MSMEG∆*fur*B with pMP1102, harbouring the complete *mpt* cluster and the 5’UTR of *mpt*A. qRT-PCR analyses revealed that the expression level of *mpt*A in MSMEG∆*fur*B was significantly higher compared to MSMEGwt at standard culture conditions (Figure 3B), suggesting a loss of repression by FurB. In addition, analysis of β-galactosidase activity in MSMEG∆*fur*B transformed with pJEM-*mpt*A2, harbouring the functional Zur box3, showed a complete derepression of promoter activity (Figure 3C). Addition of TPEN did not increase *mpt*A promoter activity in the β-galactosidase assay (Figure 3C) or gene expression in the MSMEG∆*fur*B pMP1102 transformant, analysed by qRT-PCR (data not shown). Thus, these data strongly suggest a FurB-dependent regulation of the *mpt*ABC operon in MAP.

### Transcriptional response of MAP to zinc starvation

Since the *mpt*ABC and *sid* operons are part of the MAP specific genomic insertion LSP14, we were interested to investigate the overall response of genes of this and other LSPs to zinc starvation. For this purpose, we performed RNA deep sequencing of RNA from untreated and TPEN treated MAP cultures and dissected differential gene expression by Rockhopper analysis (Additional file 1: Table S3). In total, 70 genes were found to be at least >3-fold differentially expressed in the TPEN culture compared to the untreated control (Table 1).

**Table 1:** **Zinc dependent differentially expressed genes**

		Orthologue genes (% similarity)					
RCN ^a^	Locus tag	Mtb	MAA	q-value ^b^	Fold change ^c^	Putative function ^d^	COG ^e^
-	MAP4069c	-	MAV_4568 (99.3)	<0.0001	9.5	unknown function	S
-	MAP4065	Rv0924c (43.62)	MAV_4571 (99.8)	0.0044	3.16	putative cation-transport membrane protein, NRAMP family	P
-	*MAP3788*	Rv0292 (61.6)	MAV_4860 (98.3)	<0.0001	25.48	ESX type VII secretion protein EccE	-
-	*MAP3787*	Rv0291 (69.9)	MAV_4862 (98.9)	<0.0001	30.43	type VII secretion-associated serine protease MycP3	-
-	*MAP3786*	Rv0290 (80.1)	MAV_4863 (99.4)	<0.0001	15.38	type VII secretion integral membrane protein EccD	-
-	*MAP3785*	Rv0289 (77.0)	MAV_4864 (99.7)	<0.0001	22.69	putative ESX-3 secretion-associated protein	-
-	*MAP3784*	Rv0288 (79.0)	MAV_4865 (100)	<0.0001	22.09	Esat-6 like protein EsxH	S
-	*MAP3783*	Rv0287 (84.5)	MAV_4866 (100)	<0.0001	20.81	Esat-6 like protein EsxG	-
-	*MAP3782*	Rv0286 (41.85)	MAV_4867 (98.6)	<0.0001	22.37	PPE-family protein	N
-	*MAP3781*	Rv0285 (85.1)	MAV_4868 (99.0)	<0.0001	29.76	PE-family protein	-
-	*MAP3780*	Rv0284 (84.9)	MAV_4869 (93.47)	<0.0001	23.91	type VII secretion protein EccCa/type VII secretion protein EccCb	D
-	*MAP3779*	Rv0283 (72.6)	MAV_4870 (87.5)	<0.0001	37.19	hypothetical protein, ESX type VII secretion protein EccB,	-
-	*MAP3778	Rv0282 (86.5)	MAV_4871 (91.5)	<0.0001	28.87	putative ESX-3 type VII secretion system protein EccA	O
-	MAP3776c	Rv2059 (51.45)	MAV_0583 (46.3)	<0.0001	13.81	hypothetical protein, putative permease	P
-	MAP3775c	Rv2397c (52.38)	MAV_0582 (55.5)	<0.0001	15.26	ATPase component of Mn/Zn ABC-type transporter	P
-	MAP3774c	Rv2060 (37.0)	MAV_0581 (51.89)	<0.0001	17.58	ABC-type Mn2+/Zn2+ transport system, permease component	P
-	MAP3773c	Rv2359 (56.8)	MAV_2036 (58.06)	<0.0001	16.92	Fe2+/Zn2+ uptake regulation protein, Fur family protein	P
-	*MAP3772c	-	-	<0.0001	140.73	cobW-like cobalamin synthesis, metal chaperone	R
rpmE2	*MAP3771*	-	-	<0.0001	183.4	50S ribosomal protein L31	J
-	*MAP3770	Rv0106 (66.1)	MAV_4874 (73.5)	<0.0001	218.07	cobW-like cobalamin synthesis, metal chaperone	R
rpmG	*MAP3769c	Rv2057c (85.2)	MAV_4876 (93.5)	<0.0001	191.92	50s ribosomal protein L33	J
rpsN2	*MAP3768c*	Rv2056c (81.2)	-	<0.0001	194.0	30S ribosomal protein S14 RpsN2	J
rpsR2	*MAP3767c*	Rv2055c (77.2)	MAV_0076 (84.61)	1.0	22.32^#^	30S ribosomal protein S18	J
-	*MAP3766*	-	MAV_4878 (85.1)	<0.0001	6.83	hypothetical protein, putative permease	R
-	*MAP3765	Rv3738c (80.43)	MAV_4879 (76.1)	<0.0001	24.43	PPE-family protein	N
pks2	*MAP3764c	Rv1180 (78.67)	MAV_2370 (68.0)	<0.0001	23.31	polyketide synthase Pks2	Q
papA3_2	*MAP3763c*	Rv1182 (68.24)	MAV_2723 (66.35)	<0.0001	21.0	polyketide synthase associated protein papA3	-
-	*MAP3762c*	Rv1524 (67.83)	MAV_3994 (70.74)	<0.0001	35.58	putative glycosyl hydrolase	GC
-	*MAP3761c*	Rv1517 (50.86)	MAV_1758 (50.58)	<0.0001	5.03	unknown function	-
-	MAP3760c	Rv2952 (72.0)	MAV_3877 (50.58)	<0.0001	4.02	unknown function	H
fadD28	MAP3752	Rv3826 (61.4)	MAV_2374 (67.1)	<0.0001	3.4	acyl-CoA synthetase	IQ
mmpL4_5	MAP3751	Rv0507 (64.9)	MAV_3863 (65.4)	<0.0001	26.35	MmpL-family protein, MmpL4_5	R
mmpS1	MAP3750	Rv0451c (69.49)	MAV_3864 (68.37)	<0.0001	67.0	putative membrane protein	-
-	MAP3749	Rv2750 (49.46)	MAV_2946 (60.0)	<0.0001	163.04	3-ketoacyl-ACP reductase, caRveol dehydrogenase	IQR
IS1110	MAP3748c	Rv2177c (50.0)	MAV_1059 (43.31)	0.00012	4.42	IS1110 transposase	L
cobW	*MAP3747c	Rv0106 (59.53)	MAV_4874 (58.39)	<0.0001	41.47	putative cobalamin synthesis protein	R
sidB	*MAP3741*	Rv2383c (45.86)	MAV_2009 (66.47)	0.0	150.4^#^	putative thioester reductase	-
sidA	*MAP3740	Rv2383c (45.86)	MAV_2013 (47.41)	<0.0001	237.0	putative non-ribosomal peptide synthase, thioester reductase	Q
sidG	*MAP3739c	Rv2333c (44.2)	-	<0.0001	270.0	MFS transporter permease	G
-	*MAP3738c*	-	-	<0.0001	121.73	type12 methyltransferase	R
-	*MAP3737	Rv0280 (63.9)	MAV_4872 (59.37)	<0.0001	75.65^#^	PPE-family protein	N
mptA	*MAP3736c	Rv1348 (50.0)	MAV_1566 (51.37)	<0.0001	39.4	hypothetical protein, ABC-transporter ATPase	V
mptB	*MAP3735c*	Rv1348 (54.48)	MAV_1566 (54.16)	<0.0001	78.18	ABC transporter ATP-binding protein	V
mptC	*MAP3734c*	Rv1348 (59.33)	MAV_1566 (58.36)	<0.0001	19.0	putative ABC transporter ATPase and permease component	V
mptD	*MAP3733c*	-	-	<0.0001	5.77	unknown function	-
mptE	*MAP3732c*	-	-	<0.0001	13.14	ABC-type cobalt transport system, permease component	P
mptF	*MAP3731c*	Rv3663c (44.93)	-	<0.0001	4.11	ABC-type cobalt transport system, ATP binding component	P
-	MAP3632	Rv0190 (89.6)	MAV_4988 (100)	<0.0001	5.23	unknown function	S
-	MAP3626c	-	MAV_4993 (100)	<0.0001	29.25	metallothionein	-
-	MAP3492	Rv3401 (86.0)	MAV_4352 (99.7)	<0.0001	10.27	putative glycosyl hydrolase	G
-	MAP3491	Rv3400 (77.3)	MAV_4350 (98.9)	<0.0001	22.04	hypothetical protein, beta-phosphoglucomutase hydrolase	R
-	MAP3047	Rv3013 (88.0)	MAV_3861 (100)	<0.0001	16.0	ACT domain-containing protein	TK
-	MAP2999	Rv2963 (80.7)	MAV_3787 (99.2)	<0.0001	16.82	hypothetical protein	R
-	MAP2414c	Rv1348 (79.6)	MAV_1566 (97.7)	<0.0001	5.0	iron ABC transporter permease	V
-	MAP2412c	-	MAV_1568 (98.6)	<0.0001	6.0	phosphotransferase enzyme family protein	R
trpE2	MAP2205c	Rv2386c (74.0)	MAV_1792 (99.6)	<0.0001	4.54	salicylate synthase MbtI	EH
mbtB	MAP2177c	Rv2383c (72.1)	MAV_2009 (98.6)	<0.0001	10.0	Phenyloxazoline synthase	Q
-	MAP2176c	Rv2383c (58.02)	MAV_2010 (98.6)	<0.0001	10.0	phenyloxazoline synthase, thioesterase	Q
mbtC	MAP2175c	Rv2382c (71.9)	MAV_2011 (99.1)	<0.0001	5.75	polyketide synthase, erythronolide synthase	Q
mbtD	MAP2174c	Rv2381c (53.08)	MAV_2012 (98.2)	<0.0001	4.43	MbtD, polyketide synthase	Q
mbtE	MAP2173c	Rv2380c (74.38)	MAV_2013 (96.8)	<0.0001	4.35	MbtE, partial linear gramicidin synthetase subunit D	Q
-	MAP2172c	Rv2380c (51.11)	MAV_2013 (99.3)	<0.0001	5.58	fmnh2-utilizing oxygenase	Q
smtB	MAP2138	Rv2358 (74.4)	MAV_2037 (99.3)	0.00034	- 7.5	ArsR family transcriptional regulator	K
-	MAP1977c	Rv0560c (43.04)	MAV_2216 (98.6)	<0.0001	12.5	CheR methyltransferase, SAM binding domain	R
-	MAP1555c	Rv1344 (77.8)	MAV_2873 (100.0)	<0.0001	7.81	acyl carrier protein	IQ
fadD33_2	MAP1554c	Rv1345 (70.0)	MAV_2874 (99.2)	<0.0001	3.67	acyl-CoA synthetase	IQ
fadE14	MAP1553c	Rv1346 (81.9)	MAV_2876 (99.2)	<0.0001	3.99	acyl-CoA dehydrogenase FadE14	I
-	*MAP0489c	Rv2059 (63.1)	MAV_0583 (99.0)	<0.0001	27.14	putative Zinc ABC transporter, periplasmic-binding protein ZnuA	P
-	*MAP0488c*	-	MAV_0582 (99.3)	<0.0001	30.0	putative Zinc ABC transporter, ATP-binding protein ZnuC	P
-	*MAP0487c*	Rv2060 (84.2)	MAV_0581 (99.7)	<0.0001	10.07	putative Zinc ABC transporter, transmembrane protein ZnuB	P

^a^Reference Common Name.

^b^q-value of differentially expressed genes MAPwt standard culture vs. MAPwt TPEN culture calculated by Rockhopper analysis. A q-value < 0.01 is considered as significant.

^c^gene expression values of MAPwt TPEN culture divided by gene expression values of MAPwt standard culture from RNA-sequencing.

^d^Putative function based on Blast2Go or NCBI blastx analysis.

^e^Functional classification of proteins has been performed by use of COG database with MAP-K10 as a reference (http://www.ncbi.nlm.nih.gov/sutils/coxik.cgi?gi=380). (C) Energy production and conversion, (D) Cell cycle control, (E) Amino acid transport and metabolism, (G) Carbohydrate transport and metabolism, (H) Coenzyme transport and metabolism, (I) Lipid transport and metabolism, (J) Translation, (K) Transcription, (L) Replication, recombination and repair, (N) Cell motility, (O) Posttranslational modification, protein turnover, chaperones, (P) Inorganic ion transport and metabolism, (Q) Secondary metabolites biosynthesis, transport and catabolism, (R) General function prediction only, (S) Function unknown, (T) Signal transduction mechanisms, (V) Defense mechanisms.

*genes preceded by predicted FurB binding sites from FIMO analysis.

^#^zinc dependent expression was confirmed by qRT-PCR.

Clustering of the differentially expressed genes into orthologous groups (COG) revealed that 35% of the TPEN responsive genes are involved in inorganic ion transport and metabolism (P) as well as secondary metabolites biosynthesis, transport and catabolism (Q) and defence mechanisms (V).

A high number of genes (54) was organized in 16 operons and most interestingly, 28 differentially expressed genes were located on LSP14 and LSP15. All genes except one were higher expressed in the TPEN treated culture. The single lower expressed gene was *smt*B (*map*2138), a zinc-sensing repressor, which is de-repressed when Zn^2+^ is available [4]. The group of higher expressed genes was comprised of ABC-type metal transporters and systems, including the *mpt* cluster and parts of the *sid* operon (*map*4065, *map*3774c-3776c, *map*3731c-3736c, *map*3739c-3741, *map*2414c, *map*0487c-0489c), as well as an ESX-typeVII secretion system and PE-/PPE-family proteins (*map*3778-3788, *map*3765, *map*3737). Also, the mycobactin clusters *mbt*1 (*map*2172c-2177c) and *mbt*2 (*map*1553c-1555c) were slightly induced by zinc starvation. Furthermore, induction of genes encoding for ribosomal proteins (*map*3771, *map*3767c-3769c) and *cob*W-like chaperons (*map*3772c, *map*3770c, *map*3747c) was observed.

FIMO analysis based on the Zur box motif of Mtb revealed the presence of 19 putative Zur boxes (Table 2) in the 5’ region of 12 regulated genes or gene clusters (Table 1 asterisks) of the MAP zinc regulon, in toto comprising 38 genes (Table 1 italics).

**Table 2:** **Zur boxes of the MAP zinc regulon as predicted by FIMO analysis**

Locus tag	RCN ^a^	Position ^b^	Zur box ^c^
MAP3778		−236	TGATAATGAAAATGATTTTCGTTA
MAP3772c		−30	CGTTAATGAAAATGATTATCATTA
MAP3770		−88	GCTTATTGAAAATGATTTTCGACA
		−33	TCGAGATGAAAATGATTCCCAATA
MAP3769c	rpmG	−283	TCTTGTCGAAAATCATTTTCAATA
		−338	CCTTATTGGGAATCATTTTCATCT
MAP3765		−270	GCTTACTGAAAATGATTGTTATTA
		−139	TGTTAACGAAAATCGTTTTCAGTA
MAP3764c	pks2	−307	GTTTACTGAAAACGATTTTCGTTA
MAP3747c	cobW	−88	GCTTATTGAAAACGATTTTCGACA
		−33	GCTAGATGAAAACGATTGTCGATA
MAP3740	sidA	−118	CGACAATGAAAATCGTTTTCAGTA
MAP3739c	sidG	−21	CCTTACTGAAAACGATTTTCATTG
MAP3737		−197	CCGTAATGAAAATGATTTTCATTA
		−248	GGTTATTGAAAATGACTTTCATTA
MAP3736c	mptA	−65^d^	TGTTATCGATAACGATTTTCATTA
		−440^d^	CTTTAATGAAAGTCATTTTCAATA
		−491^d^	CCGTAATGAAAATCATTTTCATTA
MAP0489c		−66	TTGTAATGGAAACGATTTTCATTA

^a^Reference Common Name

^b^Position of the first base relative to the annotated translation start site [NCBI:NC_002944].

^c^Putative Zur binding sites according to MEME-SUITE and FIMO analysis on the basis of MAP K-10 genome [NCBI:NC_002944].

^d^Position of the first base relative to the transcription start site determined by RACE experiments.

**Figure 4:**
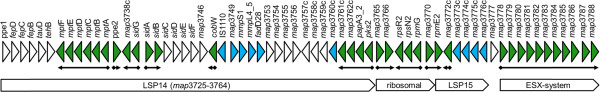
**Organisation of a** ***M. avium*** **ssp.** ***paratuberculosis*** **specific zinc responsive genomic island (ZnGI).** Depicted are the genes *map*3725 to *map*3788. Genes responsive to zinc starvation are colored either blue (no Zur box) or green (Zur box). Location of Zur boxes and genes under their control are marked by black arrows. LSP14, LSP15 and two other gene clusters are marked by white bold arrows at the bottom.

Remarkably, with the exception of one gene cluster, all genes preceded by a putative Zur box were clustered on a single 90 kb gene locus from *map*3725 to *map*3788 (64 genes) spanning LSP14 and LSP15 (Figure 4), hereafter designated as MAP specific zinc responsive genomic island (ZnGI). Only the operon with MAP homologues to the well described zinc transporter ZnuABC (*map*0487c-0489c) harboured a Zur box in the 5’ region and was encoded elsewhere in the genome.

Apart from putative Zur-regulated genes, we also found a number of zinc dependent but Zur-independent genes, either distributed over the genome (22 genes) or located on the 90 kb gene locus (10 genes).

To proof the significance of the zinc responsive gene clustering on the ZnGI in MAP, a Monte-Carlo simulation of non-random gene distribution was performed. In 84.2% of the simulated data sets, four or five zinc responsive genes out of 70 randomly distributed ones were found to be clustered within 64 genes. The largest cluster consisting of nine zinc responsive genes was found in one data set only. Based on these results, the probability that 45 out of 70 zinc responsive genes are located in a region of 64 out of 4,350 genes by chance is *p* < 1.0*10^−4^.

Finally we analysed the genomic distribution of homologues to zinc responsive genes of MAP and additional Zur dependent genes of Mtb in other mycobacteria species (Mtb, MSMEG, MAA, *M. bovis* BCG Pasteur, *M. marinum*, *M. leprae*, *M. vanbaalenii*) (Additional file 1: Table S4). Interestingly, in contrast to MAP, we found no evidence for a locus specific clustering of these genes in the other mycobacterial species, demonstrating the specificity of the ZnGI for MAP.

## Discussion

In contrast to other mycobacteria, MAP exhibits a strong tropism to the gut, but the molecular mechanisms which enable MAP to fill this niche are only poorly understood. The lineage specific genomic insertions LSP14 and LSP15, unique to MAP and acquired during evolution, are suggested to be involved in metal homeostasis and have been found to be associated with virulence [18–20, 32]. Since acquisition of metals is a crucial pathomechanism, in the present study we intended to analyse the metal dependent regulation of the LSP14-15 gene locus. Initially, our particular interest was drawn to the *mpt* (**m**ycobacterium **p**aratuberculosis **t**ransporter) cluster, comprised of two predicted transporters (*mpt*ABC and *mpt*DEF), as it might represent an alternative iron scavenging system, which possibly compensates for MAP defects to produce mycobactins [31]. In fact, *mpt*ABC was hypothesised to represent the iron regulated transporter IrtAB in MAP [40]. However, our starvation experiments clearly showed a sensitive regulation of the *mpt*ABC operon by zinc but not by iron.

Zinc homeostasis is tightly regulated by the FUR family regulator FurB (Zur) in many bacteria [41, 42], as zinc starvation as well as zinc excess ultimately lead to cell death. Despite this, only little is known about zinc homeostasis in mycobacteria. Analyses in Mtb indicated the regulation of zinc transporters and storage systems by Zur [10]. Yet, functional studies are missing so far and no research has been conducted on MAP.

Our *in silico* analyses revealed that FurB of MSMEG, Mtb and MAP are almost identical and share the same structural and catalytic amino acids. In addition, by FIMO analyses we found a conserved homology of MAP Zur boxes to Zur boxes of Mtb and other bacteria (Table 2 [10, 43])*.* In fact, by site directed mutagenesis, we could demonstrate the specificity of these boxes. Mutation of two single, highly conserved nucleotides [**G**ANAANNNTTTT**C**] in the FurB binding site located in the 5’ UTR of the *mpt*ABC operon (Zur box3) resulted in the loss of gene repression in MSMEG.

Using MSMEG as a heterologous model, we showed a highly sensitive transcriptional response of the *mpt*ABC operon to zinc starvation. In addition, by generating a MSMEG∆*fur*B mutant, we were able to show that FurB is involved in *mpt*ABC regulation. Deletion of FurB in MSMEG led to a complete loss of *mpt*A gene repression. This clearly demonstrated the importance of FurB in *mpt*ABC regulation and implies a putative role of *mpt*ABC as zinc importer.

RNAseq allowed us to assess the global response of MAP to zinc limitation and provided an interesting insight in MAP zinc homeostasis. Overall the majority of the 70 differentially expressed genes could be assigned to metal homeostasis. Notably, 45 zinc responsive genes were clustered on a single 90 kb locus in the genome, spanning the region from *map*3725 to *map*3788 (Figure 4). Therefore, we specified this locus as MAP specific zinc responsive genomic island (ZnGI).

11 predicted Zur box containing promoter-operator elements located on the ZnGI suggest the control of in toto 35 genes by Zur. The ZnGI comprises LSP14 (*map*3725-3764c), an intermediate cluster with a high number of genes for ribosomal proteins (*map*3765-3770), LSP15 (*map*3771-3776c) and the adjacent gene cluster *map*3778-3788 (Figure 4). The latter is preceded by a Zur box and encodes for an ESX-typeVII secretion system, which mediates the transport of virulence associated PE and PPE proteins [44]. In addition, it was shown to be necessary for iron and zinc metabolism in Mtb [45] as well as host-pathogen interaction [46]. Also the ZnGI genes *map*3771, *map*3769c, *map*3768c, and *map*3767c encoding for the ribosomal proteins *rpm*E2, *rpm*G2, *rps*N2 and *rps*R2, respectively, were dramatically induced upon zinc starvation. The presence of Zur binding sites upstream of the ribosomal genes in MAP suggested suppression by MAP^Zur^. These genes are paralogues of the genes *rpm*E1, *rpm*G1, *rps*N1 and *rps*R1 (*map*2463c, *map*4106, *map*4180, *map*0069) which are localised beyond the ZnGI in the MAP genome and were not induced by zinc depletion. Interestingly, in contrast to the latter, the corresponding proteins encoded by *rpm*E2, *rpm*G, *rps*N2 and *rps*R2 do not bear zinc binding CXXC motifs, suggesting that they are functional substitutes of the others in the absence of zinc as it has been described for Mtb, *Streptomyces coelicolor* and *Bacillus subtilis* [10, 47, 48]. Moreover, the ZnGI harboured three putative Zur regulated genes of CobW like proteins, which were induced to a very high extent. Proteins of this family (COG0523) are linked to zinc homeostasis in all kingdoms of life [43] and have been found to be Zur-regulated in other bacteria [10, 49, 50]. These proteins possibly constitute low-affinity zinc transporters or chaperones, which are utilized to direct Zn^2+^ ions to the proper protein.

Interestingly, contrary to so far described genes, the gene cluster *map*3761c-3764c on the ZnGI, most probably regulated by Zur, was predicted to be involved in lipid and carbohydrate metabolism. Furthermore, two gene clusters lacking a predicted Zur binding site located on the ZnGI were highly induced by zinc: a group of virulence associated membrane proteins (MmpL4/MmpS1), and an enzyme involved in fatty acid biosynthesis (*map*3749-3751). Together with the zinc induced expression of the paralogous ribosomal genes, the regulation of the above groups of genes suggests that MAP to some extent changes its metabolism to adapt to zinc starvation.

Comparison of the MAP zinc regulon and the Zur regulon of Mtb [10] revealed a high congruency. Orthologues of 23 Mtb Zur regulated genes were found on the MAP ZnGI which however is in striking contrast to Mtb where the Zur regulon is organized in small clusters scattered over the genome. In addition, homologues of 9 genes of the Mtb regulon were either not present (4) or regulated (5) in MAP, indicating that other genes of the ZnGI may substitute their functions. Analysis of homologue genes and cluster analysis in other pathogenic and apathogenic mycobacteria also confirmed the unique clustering of zinc regulated genes on the ZnGI of MAP. The presence of a ZnGI in the sheep strain MAP S397 is likely, but since there is only an incomplete genome sequence available, we can only speculate this point.

Only one predicted Zur binding site was located outside the ZnGI, preceding MAP homologues (*map*0487c-0489c) to the well described high-affinity zinc importer ZnuABC of *E. coli* [51, 52] and *Salmonella* Typhimurium [53]. Thus, MAP possesses three putative zinc responsive transporters: the ZnuABC transporter and two ZnGI located transporters, namely *mpt*ABC (*map*3736c-3734c) and an ABC-type Mn^2+^/Zn^2+^ transporter (*map*3773c-3776c). All were induced by zinc starvation (Table 1), however only the first two systems seem to be regulated by Zur. Furthermore the ABC-type Mn^2+^/Zn^2+^ transporter and the *mpt*ABC transporter have no homologues in other mycobacteria and in concert with the above described data might enable MAP to more efficiently circumvent zinc starvation. Together these data point to a very particular relevance of zinc in MAP.

Beyond the ZnGI, zinc responsive genes mainly showed a weak reaction to zinc starvation, including the mycobactin cluster *mbt*1 (*map*2172c-2177c) and *mbt*2 (*map*1553c-1555c). Gene expression was slightly increased but compared to the specific iron dependent response (Figure 1A) we consider this induction as a secondary effect of metal chelation. This might also hold true for other weakly induced genes.

## Conclusions

In summary, our data confirmed the initial assumption of an involvement of the lineage specific gene loci LSP14 and LSP15 in metal homeostasis. But other than previously assumed, they were highly responsive to zinc starvation. Moreover, we found a striking particularity for MAP zinc homeostasis, given by the significant clustering of zinc regulated genes on a large 90 kb spanning zinc specific locus, which was not found in other mycobacteria, and the presence of MAP specific zinc transporters. In general, MAP seems to be well adapted to maintain zinc homeostasis. The importance of zinc transporters in the gut for colonisation and survival in the mucosal environment has been shown for *S.* Typhimurium, *Acinetobacter baumanii* and *Campylobacter jejuni* [54–56]. Thus, presumably the MAP specific ZnGI points to particular processes of adaptation, enabling MAP to develop its unique gut tropism. However, this assumption has to be addressed in future studies.

## Methods

### Bacterial strains, chemicals and growth conditions

All chemicals were purchased from Sigma-Aldrich (Munich, Germany) if not stated otherwise. Strains and plasmids used in this study are listed in Additional file 1: Table S1, primers in Additional file 1: Table S2. *Escherichia coli* DH5αF’ was grown in Luria-Bertani (LB) broth or LB-agar supplemented with 50 μg/ml kanamycin, 100 μg/ml hygromycin or 100 μg/ml ampicillin if necessary. Liquid cultures were incubated at 37°C in a shaking incubator at 200 rpm. Competent cells were prepared as described earlier [57]. *E. coli* DH5αF’ cells were used for the construction of plasmids for reporter assays and deletion mutants. *Mycobacterium avium* ssp. *paratuberculosis* strain DSM 44135 (MAP) was grown in Difco™ Middlebrook 7H9 medium or on Middlebrook 7H10 agar (Beckton Dickinson, Franklin Lakes, NJ, USA) supplemented with 10% OADC (0.06% oleic acid, 5% albumin, 2% dextrose, 0.085% NaCl, 0.003% catalase), mycobactin J (2 mg/l, Allied Monitor) and 2.5% glycerol, following referred to as MB-complete. Liquid cultures were incubated either in a shaking incubator at 100 rpm or in a stirring bottle at 150 rpm / 37°C to an OD_600_ of 1.0. If required, kanamycin or hygromycin were added to a final concentration of 50 μg/ml.

*Mycobacterium smegmatis* mc^2^ 155 (MSMEG), *M. smegmatis*∆*fur*B (MSMEG∆*fur*B) and transformed strains were grown in MB-complete, supplemented with kanamycin or hygromycin (50 μg/ml), if necessary. Liquid cultures were incubated at 37°C in a shaking incubator at 150 rpm. MSMEG competent cells were prepared according to Parish & Stoker [58].

For metal starvation, MAPwt or MSMEG strains were cultivated in MB-complete to an OD_600_ of 1.0; then the cultures were divided and either treated with 2,2’-bipyridyl (DIP, 200 μM final), or nitrilotriacetic acid trisodium salt (NTA, 14 mM final), or *N,N,N',N'*-tetrakis (2-pyridylmethyl) ethylenediamine (TPEN, 10 μM final) with mild agitation. If appropriate, cultures were supplemented with ZnSO_4,_ FeSO_4_, MgCl_2_, CaCl_2_, CuSO_4_, CoCl_2_ or MnSO_4_ to a final concentration of 1 mM for NTA treated cultures or of 7.5 μM for TPEN treated cultures. Cells were harvested at the indicated time points and subjected to further analysis.

MSMEG transformants for reporter assays were grown in MB-complete to an OD_600_ of 1.0. Cultures were split and incubated 2 h with or without 10 μM TPEN in a shaking incubator at 37°C and 150 rpm. Subsequently, cells were harvested, lysed and extracted proteins were subjected to β-galactosidase assays.

### Extraction of nucleic acids

Genomic DNA was extracted as published earlier [32]. For preparation of plasmids NucleoBond® AX kit was used (Macherey and Nagel GmbH, Düren, Germany) according to the manufacturer’s protocol.

Total RNA from cell pellets obtained in metal starvation experiments was isolated using the RNeasyMINI kit (Qiagen, Düsseldorf, Germany) according to the manufacturers protocol with minor modifications as described earlier [19, 59]. The RNA was treated twice with 50 U of DNase I (Roche, Mannheim, Germany) and subsequently purified using the RNeasyMINI kit. Quality of RNA was confirmed by agarose gel electrophoresis and spectrophotometric analysis (Biotek, Bad Friedrichshall, Germany) at 260 nm.

### cDNA synthesis and quantitative real-time PCR (qRT-PCR)

First strand synthesis of DNAse digested RNA and subsequent qRT-PCR experiments were performed as described earlier [19]. In brief 4 μg DNase treated total RNA was subjected to cDNA synthesis using random primers (Promega, Madison, WI, USA), diluted with 90 μl ddH_2_O and 2.5 μl of each sample was used for qRT-PCR. Efficacy of qRT-PCR primers (Additional file 1: Table S2) was tested with serial dilutions of genomic DNA. All samples were analysed in duplicate, results were normalized to the housekeeping gene *gap* (*map*1164 or *msmeg*3084) and expressed as fold-change compared to the untreated control.

### Rapid amplification of 5’-cDNA ends (5’RACE)

Determination of the transcriptional start point of *mpt*A was performed by 5’RACE® (Invitrogen, Life Technologies, Darmstadt, Germany), using cDNA synthesized from RNA of TPEN treated cultures with gene specific primer ocDNAmptA. Briefly, RNA was treated with Terminator 5’-Phosphate-Dependent Exonuclease (TEX, Epicentre, Madison WI, USA) prior cDNA synthesis, to digest degraded mRNA transcripts. Following, an oligo-dC tail was attached by using terminal deoxynucleotidyl transferase (TdT, Invitrogen, Life Technologies, Darmstadt, Germany). The tailed cDNA was amplified by use of a nested gene specific primer (oGSP1mptA) and 5’ RACE® Abridged Anchor primer (AAP). PCR products were cloned into pJet™1.2 (ThermoFisher Scientific, Waltham, MA, USA) and plasmids of three transformants were submitted to sequencing (Seqlab, Göttingen, Germany).

### RNA deep sequencing and analysis

To address zinc dependent gene regulation in MAP we performed RNA deep sequencing technique using 50 bp single-ends sequencing on a HiSeq2500 (Illumina, San Diego, CA). The sequence output was mapped against the genome sequence of the reference strain MAP K-10 [NCBI:NC_002944] using BWA v. 0.7.5 and SAMtools for storing nucleotide sequence alignments. Data were subsequently computed with Rockhopper tool (Additional file 1: Table S4). Genes with a q-Value <0.01 were considered as significantly differentially expressed. In some cases inconsistent expression values in the Rockhopper analyses were confirmed by qRT-PCR as described above.

### Bioinformatics and statistics

Putative functions of differentially expressed genes identified by Rockhopper analysis were identified with Blast2Go tool and NCBI blastx analysis. To analyse putative FurB binding sites in MAP [NCBI:NC_002944], a FIMO analysis was performed. Published Mtb-Zur binding sites of Rv0106, Rv2069, rpmB2, rpmB1, Rv3017c and Rv3019c [10] were used to generate the consensus sequence of Zur by MEME SUITE [CG]C[TCG]T[AG][TA][TC]GA[AT]AA[TC][ACG][AG]TT[TG][TC]C[AG][TA][TC]A (Figure 2C). This sequence was subsequently submitted to FIMO analysis. Nucleotides in brackets are variable, single nucleotides are conserved. The genomic location of detected binding sites was determined and considered as putative Zur box within −500 nucleotides relative to predicted translation start sites (TLS).

Comparison of different FurB amino acid sequences was performed with Clustal Omega. Data of qRT-PCR experiments and β-galactosidase assays are expressed as mean ± SEM. Statistical analyses were performed using either the nonparametric *t*-test (Mann–Whitney) or 1way ANOVA test (Kruskal Wallis) with GraphPad Prism 5.03 (GraphPad, San Diego, CA, USA) software.

A non-random distribution of zinc responsive genes was tested with the Monte Carlo method based on the approach of Ramachandran et al. [60]. A simulation was programed that created a data set with 4,350 genes, of which 70 were selected randomly as zinc responsive. In a second step, the maximum number of zinc responsive genes within a range of 64 genes was determined. These processes were repeated 10,000 times. Afterwards, the relative frequency of simulated zinc responsive genes within 64 genes greater or equal to the observed value was the basis for the calculation of the *p*-value. The simulation program was written in SAS, Version 9.3 TS Level 1 M3 [61].

### Cluster analysis

Homologous genes to the MAP zinc regulon and to Zur regulated genes of Mtb of different mycobacterial species were analysed for clustering. Genomes of MSMEG [NCBI:NC_008596], Mtb [NCBI:NC_000962], MAA [NCBI:NC_008595], *M. bovis* BCG Pasteur [NCBI:NC_008769], *M. marinum* [NCBI:NC_010612], *M. leprae* [NCBI:NC_011896] and *M. vanbaalenii* [NCBI:NC_008726] were compared to MAP [NCBI:NC_002944] or Mtb [NCBI:NC_000962] with the tool “Genome Genes Best Homologues” of Integrated Microbial Genomes Expert Review (IMG/ER) (https://img.jgi.doe.gov/cgi-bin/er/main.cgi) or analysed by single gene comparison via NCBI blastx analysis (min. 60% coverage and 40% identity on protein level). Following, the species specific genomic distribution of the gene homologues was evaluated by their locus tags (Additional file 1: Table S4).

### Construction and selection of a *M. smegmatis*∆*fur*B mutant

A markerless *M. smegmatis*∆*fur*B mutant was constructed using the two-step system with p2NIL and pGOAL19 plasmids [62], (Addgene plasmids 20188 and 20190). Flanking regions up- and downstream of *fur*B (*msmeg*4487) [NCBI:NC_008596] were amplified from genomic DNA of MSMEG (size 1500 bp) using primer pairs oMSMEG4487-A fw/rev and oMSMEG4487-B fw/rev by standard PCR with Phusion® High-Fidelity DNA polymerase (New England Biolabs, Beverly, MA, USA), cloned into pJET™1.2 (ThermoFisher Scientific, Waltham, MA, USA) and sequenced for correct amplification to exclude mutations in adjacent genes. Plasmids harbouring the up- and downstream fragments were restriction digested with *Hind*III/*Bbs*I (A) or *Bbs*I/*Kpn*I (B) and subsequently ligated to *Hind*III/*Kpn*I digested p2NIL [62], resulting in p2NIL-MSMEG4487-AB. *Pac*I digested marker gene cassette from pGOAL19 was ligated into p2NIL-MSMEG4487-AB resulting in p2NIL-MSMEG4487-Del. 500 ng of plasmid, pretreated with 100 mJ UV light cm^−2^, was electroporated into MSMEG electro-competent cells and selection of mutants was performed according to Parish and Stoker [62] with minor modifications. In brief, blue kanamycin resistant colonies were inoculated in 3 ml LB without antibiotic and incubated for 24 h. 2% sucrose was added and cultures were allowed to grow for 3 h under selection pressure. Aliquots of sucrose treated cells were plated on LB with 2% sucrose and X-Gal (50 μg/ml). White colonies were replica plated on LB and LB-kan. Kanamycin sensitive clones were screened by PCR with primers oMSMEG_4487-Del fw/rev.

### Construction of plasmids for β-galactosidase activity assays

Genomic DNA of MAP DSM44135 was used to amplify full and truncated 5’UTR of *mpt*A with Phusion® High-Fidelity DNA polymerase using primer pairs oRep-JEM-fw/-rev2, oRep-JEM-fw6/-rev2, oRep-JEM-fw/-rev3, respectively (restriction enzyme sites underlined, Additional file 1: Table S2). Fragments were digested with *Sca*I/*Bam*HI and cloned into pJEM15 [63], resulting in plasmids pJEM-*mpt*A2, harbouring the whole 5’UTR of *mpt*A without the ribosome binding site, pJEM-*mpt*A8 with Zur box3 only and 295 bp upstream this box and pJEM-*mpt*A3 lacking Zur box3 and putative promoter elements. Mutation of a predicted FurB binding site was achieved by inverse site directed mutagenesis PCR on plasmid pJEM-*mpt*A2 using Phusion® polymerase and primers o*mpt*A2-JEM-Mut-fw/rev with two single point mutations in the forward primer (mutated bases underlined, Additional file 1: Table S2) resulting in vector pJEM-*mpt*A2MUT. Correct construction of plasmids was determined by restriction enzyme digestion and sequencing. All plasmids were transformed into MSMEGwt and/or MSMEG∆*fur*B. Functionality of promoter and putative FurB binding sites were analysed by β-galactosidase activity assay.

### β-Galactosidase activity assay

*M. smegmatis* strains harbouring the indicated reporter plasmids were grown in MB-complete to an OD_600_ of 1.0 and treated with 10 μM TPEN as described above. Subsequently, protein extraction was conducted as follows: 100 mg of wet cell pellet was resuspended in 50 mM Tris–HCl buffer (pH 7.5); protease inhibitor (AEBSF) was added to a final concentration of 500 μM. Suspension was transferred to a tube containing 300 mg circonium beads and cells were disrupted in a bead beater (level 6) 3 times for 30 sec with 5 min intermediate cooling steps. Lysates were then transferred to reaction tubes and subsequently sonicated at 4°C for 20 min (duty cycle 50%). Cell debris was removed by centrifugation (11,000 × g/5 min/4°C). Protein concentration of lysates was determined with MicroBCA Protein Kit (Interchim, Montluçon, France). Lysates were diluted in Z-buffer (0.2 M Na_2_HPO_4_, 20 mM KCl, 2 mM MgSO_4_, 50 mM β-mercaptoethanol, pH 7.5) to appropriate concentrations and incubated with 40 μM *o*-nitrophenyl-*β*-D-galactopyranoside (ONPG, Sigma-Aldrich) for 45 min at 37°C. Absorption was measured at 405 nm in a fluorescence reader (Tecan GENios Pro, Männedorf, Swiss) and activity was calculated as fluorescence 405 nm/protein mg/ml.

## Availability of supporting data

The data sets supporting the results of this article are available in the European Nucleotide Archive repository, http://www.ebi.ac.uk/ena/data/view/PRJEB7826.

## Electronic supplementary material

Additional file 1: **Table S1.** Bacterial strains and plasmids. **Table S2.** Oligonucleotides. **Table S3.** Raw data of Rockhopper analysis. **Table S4.** Homologue zinc responsive genes in mycobacteria. **Figure S5.** TPEN-Zn titration experiment. (XLSX 441 KB)
